# Mutant prevention concentrations, *in vitro* resistance evolution dynamics, and mechanisms of resistance to imipenem and imipenem/relebactam in carbapenem-susceptible *Klebsiella pneumoniae* isolates showing ceftazidime/avibactam resistance

**DOI:** 10.1128/aac.01120-24

**Published:** 2024-11-15

**Authors:** Tania Blanco-Martín, Lucía González-Pinto, Pablo Aja-Macaya, Salud Rodríguez-Pallares, Lucía Sánchez-Peña, Eva Gato, María del Carmen Fernández-López, Michelle Outeda-García, Arianna Rodríguez-Coello, Rosa Pedraza-Merino, Isaac Alonso-García, Juan Carlos Vázquez-Ucha, Luis Martínez-Martínez, Jorge Arca-Suárez, Alejandro Beceiro, Germán Bou

**Affiliations:** 1 Servicio de Microbiología and Instituto de Investigación Biomédica A Coruña (INIBIC), Complexo Hospitalario Universitario A Coruña, A Coruña, Spain; 2 Unidad de Microbiología, Hospital Universitario Reina Sofía e Instituto Maimónides de Investigación Biomédica de Córdoba (IMIBIC), Córdoba, Spain; 3 CIBER de Enfermedades Infecciosas (CIBERINFEC), Instituto de Salud Carlos III, Madrid, Spain; 4 Departamento de Química Agrícola, Edafología y Microbiología, Universidad de Córdoba, Córdoba, Spain; University of Fribourg, Fribourg, Switzerland

**Keywords:** imipenem, imipenem/relebactam, KPC, Ω-loop, ceftazidime/avibactam, evolution, beta-lactamase, antimicrobial resistance, carbapenem

## Abstract

*Klebsiella pneumoniae* carbapenemase (KPC) variants selected during ceftazidime/avibactam treatment usually develop susceptibility to carbapenems and carbapenem/β-lactamase inhibitors, such as imipenem and imipenem/relebactam. We analyzed imipenem and imipenem/relebactam single-step mutant frequencies, resistance development trajectories and differentially selected resistance mechanisms using two representative *K. pneumoniae* isolates that had developed ceftazidime/avibactam resistance during therapy (ST512/KPC-31 and ST258/KPC-35). Mutant frequencies and mutant prevention concentrations were measured in Mueller–Hinton agar plates containing incremental concentrations of imipenem or imipenem/relebactam. Resistance dynamics were determined after incubation for 7 days in 10 mL MH tubes containing incremental concentrations of each antibiotic or combination, up to 64 times their baseline MIC. Two colonies per strain from each experiment were characterized by antimicrobial susceptibility testing and whole genome sequencing. The impact of KPC variants identified in resistant mutants on β-lactam resistance was investigated by cloning experiments. Imipenem/relebactam suppressed the emergence of resistant mutants at lower concentrations than imipenem, slowed down resistance development for both strains, and the resulting mutants yielded lower MICs of carbapenems and carbapenem/β-lactamase inhibitors than those selected with imipenem alone. Characterization of resistant mutants revealed that imipenem resistance was mainly caused by inactivation of OmpK36 and mutations in the KPC β-lactamase. Imipenem/relebactam-resistant mutants also maintained OmpK36 alterations, but mutations in KPC were much less frequent compared with those selected with imipenem alone. Genetic and biochemical characterization of the KPC derivatives identified in the resistant mutants confirmed their role in carbapenem resistance. Our data positions imipenem/relebactam as an attractive therapeutic option for combating ceftazidime/avibactam-resistant KPC-producing *K. pneumoniae* infections.

## INTRODUCTION

Treatment of KPC-producing *Klebsiella pneumoniae* infections poses one of the most widely recognised therapeutic challenges regarding Gram-negative bacteria. This results from the broad spectrum and increased hydrolytic activity of KPC carbapenemases, which confer high levels of resistance to all conventional β-lactam antibiotics, including carbapenems (1). In 2015, the cephalosporin/β-lactamase inhibitor combination ceftazidime/avibactam became available for clinical use, thus partly alleviating the shortage of agents available to combat infections caused by KPC-producing Enterobacterales. This combination usually demonstrates activity rates higher than 95% against KPC-producing Enterobacterales isolated in most surveillance studies and has therefore become the standard of care in settings with a high incidence of infections caused by *bla*_KPC_-positive strains (2). However, in recent years, the increased use of ceftazidime/avibactam worldwide has let to a plethora of reports of therapeutic failure and development of resistance during treatment of KPC-producing *K. pneumoniae* infections (3). The most commonly reported mechanism of resistance involves the selection of insertions, deletions, or amino acid substitutions that affect or interact within the catalytic pocket of the KPC enzyme, leading to structural rearrangements in the β-lactamase architecture that accelerate ceftazidime turnover rates and confer resistance to the ceftazidime/avibactam combination (4). To date, a wide variety of KPC derivatives associated with ceftazidime/avibactam resistance have been described, the so-called KPC variants. Most of these derivatives display substitutions within a highly mobile and flexible region designated the Ω-loop, with substitutions L169P or D179Y among the most frequently encountered (5). Recently, these kinds of variants have also been associated with cross-resistance to the innovative siderophore cephalosporin cefiderocol, adding further concern to the increasing gap caused by ceftazidime/avibactam resistance (6).

However, a feature of major therapeutic interest that is associated with treatment-emergent ceftazidime/avibactam-resistant phenotypes in Enterobacterales due to selection of KPC substitutions is the loss of the natural carbapenemase activity of the parental KPC enzyme. This effect is known as collateral susceptibility and results in the total restoration of susceptibility to carbapenems, particularly imipenem (7). This evolutionary trade-off has raised interest in the potential use of carbapenems as an attractive rescue therapy when ceftazidime/avibactam resistance emerges during treatment of KPC infections. However, previous use of carbapenems in monotherapy (or associated with other non-β-lactam agents) has produced inconsistent results, with reports of either clinical success or therapeutic failure due to re-emergence of the baseline carbapenem-resistant phenotype (8, 9). Thus, novel treatments are required to complete the armamentarium for combating infections by KPC-producing strains that become refractory to ceftazidime/avibactam.

Fortunately, in recent years, we have witnessed the introduction in the clinical setting of imipenem/relebactam, a carbapenem/β-lactamase inhibitor combination with demonstrated efficacy against MDR Gram-negative infections in two previous randomized clinical trials (RESTORE-IMI 1 and RESTORE-IMI 2) (10, 11). Relebactam is a new diazabicyclooctane inhibitor with a chemical scaffold very similar to that of avibactam but equipped with a piperidine at the 2-position carbonyl group that provides strong coverage against class A and C β-lactamases (12). More specifically, relebactam has proven to be a very potent KPC inhibitor, including of the aforementioned ceftazidime/avibactam-resistant Ω-loop variants, as demonstrated by very low *K*_iapp_ values (13). Taking advantage of the potent inhibitory activity of relebactam against both wild-type and mutated KPC enzymes, and the increased carbapenem susceptibility found in the latter, we thus hypothesize that the imipenem/relebactam combination may be a very promising option against KPC-producing strains that have become refractory to ceftazidime/avibactam. However, microbiological and clinical data regarding the potential benefit of imipenem/relebactam versus imipenem alone in combating these challenging strains have not yet been evaluated.

Thus, in order to anticipate and predict future antibacterial strategies aimed at combating resistance to ceftazidime/avibactam in KPC-producing *K. pneumoniae*, we conducted a comparative analysis of imipenem and imipenem/relebactam single-step mutant frequencies, resistance development trajectories, and differentially selected resistance mechanisms in two clinical *K. pneumoniae* isolates with distinctive KPC Ω-loop mutations associated with ceftazidime/avibactam resistance and carbapenem susceptibility.

## RESULTS AND DISCUSSION

### Suppression of single-step resistant mutants

The frequency of emergence of mutants in both of the parental strains of ceftazidime/avibactam-resistant KPC-producing *K. pneumoniae* is summarized in Table 1. Both ST512/KPC-31 and ST258/KPC-35 isolates exhibited susceptibility to carbapenems, with MICs of 0.125 and 0.5 mg/L for imipenem and 0.125 and 0.125 mg/L for imipenem/relebactam, respectively. Mutant prevention concentrations of imipenem were in all cases 8 mg/L. On the other hand, imipenem/relebactam concentration needed to suppress the emergence of resistant mutants was 2 mg/L for ST512/KPC-31 and 4 mg/L for ST258/KPC-35. Thus, despite very similar baseline MICs for both agents in the two strains evaluated, the mutant prevention concentrations of imipenem were between 64- and 16-fold higher than the imipenem MIC and in both cases higher than the EUCAST imipenem clinical breakpoint for Enterobacterales (R > 4 mg/L). Similarly, imipenem/relebactam suppressed the emergence of resistant mutants at concentrations between 16- and 32-fold higher than the baseline imipenem/relebactam MIC of the parental strain, falling within the imipenem/relebactam EUCAST susceptibility range (R > 2 mg/L) for ST512/KPC-31, and for both isolates considering the CLSI breakpoint (R > 4 mg/L). Altogether, these findings indicate that imipenem/relebactam used at conventional dosing schemes (and thus expected to reach concentrations compatible with susceptibility) has a greater chance of suppressing single-step mutants than imipenem when used alone. These findings reinforce those of Bhagunde *et al*. (14), who showed, in a recent pharmacokinetic analysis, that conventional dosing schemes would achieve a target attainment for imipenem/relebactam of 4 mg/L in plasma in 100% of patients with normal renal clearance.

**TABLE 1:** Mutant emergence frequency and mutant prevention concentrations of the parental ST512/KPC-31 and ST258/KPC-35 *K. pneumoniae* clinical isolates exposed to incremental concentrations of imipenem and imipenem/relebactam^*a*^

Strain	Imipenem MIC (mg/L)	Imipenem concentration (mg/L)	Imipenem mutant frequency	Imipenem/relebactam MIC (mg/L)	Imipenem/relebactam concentration (mg/L)	Imipenem/relebactam mutant frequency
ST512/KPC-31 (KPC-3_D179Y_)	0.125	1	-	0.125	1/4	9.39×10^−8^
		2	1.29×10^−8^		2/4	≤ 2.44×10^−10^
		4	6.10×10^−10^		4/4	-
		8	≤ 2.44×10^−11^		8/4	-
ST258/KPC-35 (KPC-2_L169P_)	0.5	1	-	0.125	1/4	-
		2	8.22×10^−9^		2/4	1.53×10^−8^
		4	5.08×10^−10^		4/4	≤ 1.69×10^−10^
		8	≤ 1.69×10^−11^		8/4	-

^*a*^ MIC, minimum inhibitory concentration; -, not tested.

### Dynamics of *in vitro* resistance development

**Fig 1:**
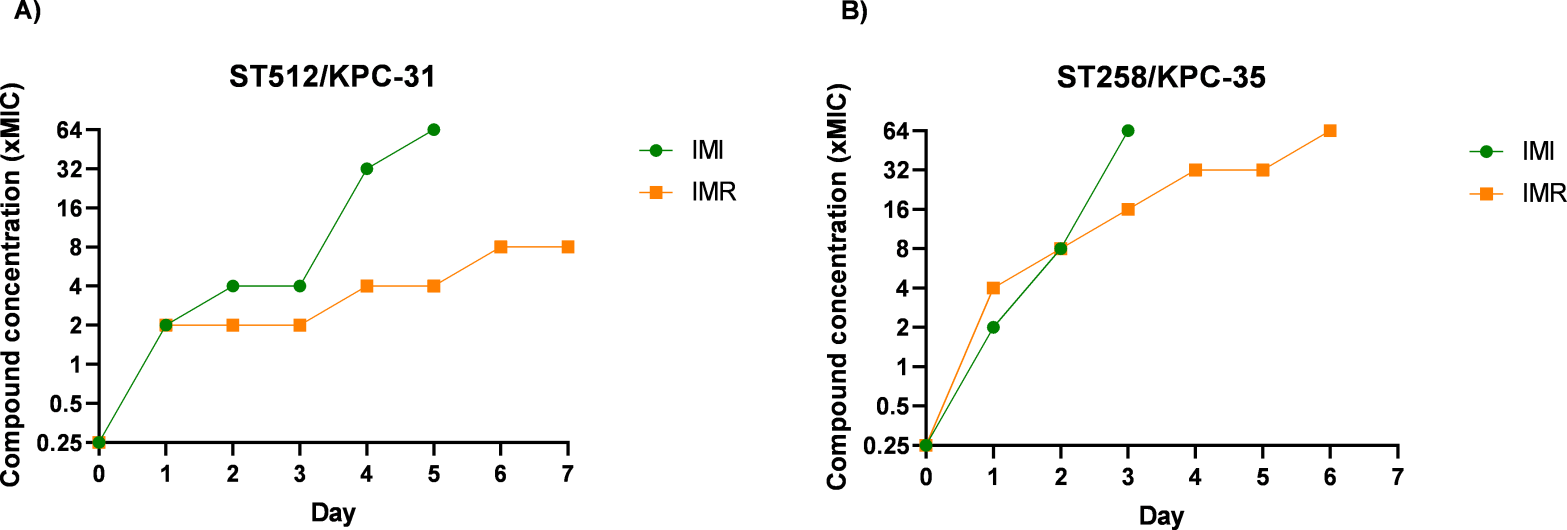
Comparative dynamics of stepwise development of resistance to imipenem and imipenem/relebactam in clinical isolates: (A) ST512/KPC-31 and (B) ST258/KPC-35. The modal values for three independent experiments are shown. IMI, imipenem; IMR, imipenem/relebactam.

Comparative analysis of the dynamics of stepwise development of resistance to imipenem and imipenem/relebactam in the ST512/KPC-31 and ST258/KPC-35 isolates is highlighted in Fig. 1, and their resistance phenotypes and genotypes are indicated in Tables 2 and 3, respectively. As commented above, at baseline, both isolates showed ceftazidime/avibactam resistance and susceptibility to imipenem and imipenem/relebactam due to the production of KPC-31 and KPC-35 (which respectively carry the Ω-loop amino acid replacements D179Y and L169P relative to the parental KPC-3 and KPC-2 enzymes, respectively) as prominent β-lactam resistance mechanisms. Both strains were also equipped with other narrow spectrum β-lactamases, either chromosomal, such as the intrinsic SHV-11, or acquired by horizontal gene transfer such as TEM-1. In addition, both had alterations in outer membrane porins that are ubiquitous in the high-risk ST258 and ST512 lineages and are associated with reduced internalization of carbapenems: a truncated OmpK35 (E42fs) and the loop three di-amino acid insertion GD in OmpK36 (GD134ins) (15). Development of imipenem resistance occurred rapidly in both strains, reaching 64× MIC values on day 5 for ST512/KPC-31 and on day 3 for ST258/KPC-35. Relebactam slowed the development of resistance in both strains, although with slight differences. Attenuation of resistance development to imipenem/relebactam was more pronounced in ST512/KPC-31, as this strain was only able to yield up to 8× MIC values after seven passages and days of experiment. For the ST258/KPC-35, the imipenem/relebactam MIC reached the maximum tested (64× MIC values), but 2 days later (day 5) than for imipenem (day 3).

**TABLE 2:** Comparative phenotypic and genotypic data of the parental ST512/KPC-31 clinical isolate and the mutants obtained after exposure to imipenem and imipenem/relebactam^,^^*d*^

Strain^*a*^			MIC (mg/L)^*b*^																	Genotype^*c*^		
			PT (R > 8)	AZT (R > 4)	AXO (R > 2)	CAZ (R > 4)	CZA (R > 8)	FEP (R > 4)	ETP (R > 0.5)	IMI (R > 4)	IMR (R > 2)	MEM (R > 8)	MV (R > 8)	SXT (R > 4)	CIP (R > 0.5)	LEV (R > 1)	GEN (R > 2)	TOB (R > 2)	AMI (R > 8)	KPC variant	Narrow-spectrum β-lactamases	Additional mutations
ST512/KPC-31			>128	16	>32	>256	64	>16	>8	0.125	0.125	4	0.5	>4/76	>2	>8	4	>8	>32	KPC-31	SHV-11, TEM-1	OmpK35 (E42fs), OmpK36 (GD134ins)
IMI	1	1.1	>128	>16	>32	>256	64	>16	>8	>256	4	>64	16	≤2/38	>2	>8	4	>8	>32	KPC-3, KPC-31	SHV-11, TEM-1	OmpK35 (E42fs), OmpK36 (Y120*), IS5 transposase (S38G), Uup (L438dup)
		1.2	>128	>16	>32	>256	64	>16	>8	>256	4	>64	8	≤2/38	>2	>8	8	>8	>32	KPC-3, KPC-31	SHV-11, TEM-1	OmpK35 (E42fs), OmpK36 (Y120*), IS5 transposase (S38G), Uup (L438dup)
	2	2.1	>128	>16	>32	>256	128	>16	>8	>256	4	>64	8	≤2/38	>2	>8	4	>8	>32	KPC-3, KPC-31	SHV-11, TEM-1	OmpK35 (E42fs), OmpK36 (Y120*), IS5 transposase (S38G)
		2.2	>128	>16	>32	>256	64	>16	>8	>256	4	>64	8	≤2/38	>2	>8	8	>8	>32	KPC-3, KPC-31	SHV-11, TEM-1	OmpK35 (E42fs), OmpK36 (Y120*), IS5 transposase (S38G)
	3	3.1	>128	>16	>32	>256	256	>16	>8	32	4	32	8	≤2/38	>2	>8	4	>8	>32	KPC-3 D179N, KPC-31	SHV-11, TEM-1	OmpK35 (E42fs), OmpK36 (GD134ins), IS5 transposase (S38G)
		3.2	>128	>16	>32	>256	256	>16	>8	32	4	32	8	≤2/38	>2	>8	4	>8	>32	KPC-3 D179N, KPC-31	SHV-11, TEM-1	OmpK35 (E42fs), OmpK36 (GD134ins), IS5 transposase (S38G)
IMR	1	1.1	>128	>16	>32	>256	128	>16	>8	4	2	16	8	≤2/38	>2	>8	4	>8	>32	KPC-31	SHV-11, TEM-1	OmpK35 (E42fs), OmpK36 (Y120*), IS5 transposase (S38G)
		1.2	>128	>16	>32	>256	256	>16	>8	2	2	16	8	≤2/38	>2	>8	4	>8	>32	KPC-31	SHV-11, TEM-1	OmpK35 (E42fs), OmpK36 (Y120*), IS5 transposase (S38G)
	2	2.1	>128	>16	>32	>256	256	>16	>8	4	2	16	8	≤2/38	>2	>8	4	>8	>32	KPC-31	SHV-11, TEM-1	OmpK35 (E42fs), OmpK36 (Y120*), HtpX (AQ86Δ)
		2.2	>128	>16	>32	>256	256	>16	>8	2	2	16	8	≤2/38	>2	>8	4	>8	>32	KPC-31	SHV-11, TEM-1	OmpK35 (E42fs), OmpK36 (Y120*), IS5 transposase (S38G)
	3	3.1	>128	>16	>32	>256	256	>16	>8	2	2	16	8	≤2/38	>2	>8	4	>8	>32	KPC-31	SHV-11, TEM-1	OmpK35 (E42fs), OmpK36 (Y120*), IS5 transposase (S38G)
		3.2	>128	>16	>32	>256	256	>16	>8	2	2	16	8	≤2/38	>2	>8	4	>8	>32	KPC-31	SHV-11, TEM-1	OmpK35 (E42fs), OmpK36 (Y120*), IS5 transposase (S38G), PurR (V271E), TraD (E591A, A607T, AS609EP)

^*a*^ Data for two colonies from each of three experiments performed with imipenem and imipenem/relebactam are shown.

^*b*^ 2024 EUCAST breakpoints for Enterobacterales indicated (v. 14.0). PT, piperacillin/tazobactam; AZT, aztreonam; AXO, ceftriaxone; CAZ, ceftazidime; CZA, ceftazidime/avibactam; FEP, cefepime; ETP, ertapenem; IMI, imipenem; IMR, imipenem/relebactam; MEM, meropenem; MV, meropenem/vaborbactam; SXT, trimethoprim/sulfamethoxazole; CIP, ciprofloxacin; LEV, levofloxacin; GEN, gentamicin; TOB, tobramycin; AMI, amikacin.

^*c*^ fs, frameshift change; ins, insertion; *, stop codon; dup, duplication; Δ, deletion.

^*d*^ Amino acid substitutions occurring in KPC enzymes were named using KPC-3 as reference. Baseline mutations were named using *K. pneumoniae* ATCC 10031 as reference.

**TABLE 3:** Comparative phenotypic and genotypic data of the parental ST258/KPC-35 clinical isolate and the mutants obtained after exposure to imipenem and imipenem/relebactam^*d*^

Strain^*a*^			MIC (mg/L)^*b*^																	Genotype^*c*^		
			PT (R > 8)	AZT (R > 4)	AXO (R > 2)	CAZ (R > 4)	CZA (R > 8)	FEP (R > 4)	ETP (R > 0.5)	IMI (R > 4)	IMR (R > 2)	MEM (R > 8)	MV (R > 8)	SXT (R > 4)	CIP (R > 0.5)	LEV (R > 1)	GEN (R > 2)	TOB (R > 2)	AMI (R > 8)	KPC variant	Narrow-spectrum β-lactamases	Additional mutations
ST258/KPC-35			128	16	>32	>256	16	>16	>8	0.5	0.125	4	1	≤2/38	>2	>8	≤2	>8	>32	KPC-35	SHV-11, TEM-1	OmpK35 (E42fs), OmpK36 (GD134ins)
IMI	1	1.1	>128	>16	>32	256	8	>16	>8	256	16	>64	16	≤2/38	>2	>8	4	>8	>32	KPC-2 L169A	SHV-11, TEM-1	OmpK35 (E42fs), OmpK36 (L32*)
		1.2	>128	>16	>32	256	8	>16	>8	256	16	>64	16	≤2/38	>2	>8	4	>8	>32	KPC-2 L169A	SHV-11, TEM-1	OmpK35 (E42fs), OmpK36 (L32*)
	2	2.1	>128	>16	>32	128	1	>16	>8	>256	4	>64	8	≤2/38	>2	>8	4	>8	>32	KPC-2	SHV-11, TEM-1	OmpK35 (E42fs), OmpK36 (GD134ins), OmpK36 (Y139*)
		2.2	>128	>16	>32	128	1	>16	>8	>256	4	>64	8	≤2/38	>2	>8	4	>8	>32	KPC-2	SHV-11, TEM-1	OmpK35 (E42fs), OmpK36 (GD134ins), OmpK36 (Y139*)
	3	3.1	>128	>16	>32	64	4	>16	>8	128	4	64	8	≤2/38	>2	>8	4	>8	>32	KPC-2 L169A	SHV-11, TEM-1	OmpK35 (E42fs), OmpK36 (GD134ins), OmpK36 (Y139*)
		3.2	>128	>16	>32	128	4	>16	>8	128	4	64	4	≤2/38	>2	>8	4	>8	>32	KPC-2 L169A	SHV-11, TEM-1	OmpK35 (E42fs), OmpK36 (GD134ins), OmpK36 (Y139*)
IMR	1	1.1	>128	>16	>32	>256	256	>16	>8	16	16	32	16	≤2/38	>2	>8	≤2	>8	>32	KPC-35	SHV-11, TEM-1	ΔOmpK36, CopA-like RNA (Nt9 C→A)
		1.2	>128	>16	>32	>256	256	>16	>8	32	16	32	16	≤2/38	>2	>8	≤2	>8	>32	KPC-35	SHV-11, TEM-1	ΔOmpK36, CopA-like RNA (Nt9 C→A)
	2	2.1	>128	>16	>32	>256	128	>16	>8	16	16	32	16	≤2/38	>2	>8	4	>8	>32	KPC-35	SHV-11, TEM-1	OmpK36 (GD134ins), OmpK36 (Y139*), RfbA (P38R), RfaH (L55Q), YqjA (LV56dup)
		2.2	>128	>16	>32	>256	128	>16	>8	16	8	32	16	≤2/38	>2	>8	4	>8	>32	KPC-35	SHV-11, TEM-1	OmpK36 (GD134ins), OmpK36 (Y139*), GadA (S26C)
	3	3.1	>128	>16	>32	256	8	>16	>8	>256	64	>64	16	≤2/38	>2	>8	4	>8	>32	KPC-2 L169T	SHV-11, TEM-1	OmpK36 (GD134ins), OmpK36 (L145fs), CopA-like RNA (Nt9 C→A)
		3.2	>128	>16	>32	256	8	>16	>8	>256	64	>64	8	≤2/38	>2	>8	4	>8	>32	KPC-2 L169T	SHV-11, TEM-1	OmpK36 (GD134ins), OmpK36 (L145fs), CopA-like RNA (Nt9 C→A)

^*a*^ Data for two colonies from each of three experiments performed with imipenem and imipenem/relebactam are shown.

^*b*^ 2024 EUCAST breakpoints for Enterobacterales indicated (v. 14.0). PT, piperacillin/tazobactam; AZT, aztreonam; AXO, ceftriaxone; CAZ, ceftazidime; CZA, ceftazidime/avibactam; FEP, cefepime; ETP, ertapenem; IMI, imipenem; IMR, imipenem/relebactam; MEM, meropenem; MV, meropenem/vaborbactam; SXT, trimethoprim/sulfamethoxazole; CIP, ciprofloxacin; LEV, levofloxacin; GEN, gentamicin; TOB, tobramycin; AMI, amikacin.

^*c*^ Mutation described in nucleotide terms in non-coding transcript. fs, frameshift change; ins, insertion; *, stop codon; dup, duplication; Δ, deletion.

^*d*^ Amino acid substitutions occurring in KPC enzymes were named using KPC-2 as reference. Baseline mutations were named using *K. pneumoniae* ATCC 10031 as reference. Mutations occurring in OmpK36 were named using ST258/KPC-35 isolate as reference.

To our knowledge, this is the first work that has comparatively analyzed the dynamics of appearance of resistance to imipenem or imipenem/relebactam in strains carrying KPC Ω-loop variants, therapeutic options that usually become active *in vitro* against KPC strains that develop resistance to ceftazidime/avibactam. In previous work, we analyzed the development of imipenem/relebactam resistance dynamics in four high-risk clones of *K. pneumoniae* producing wild-type KPC-2 and KPC-3 enzymes (16). Interestingly, we found that 64× MIC values were rapidly reached (day 3) with most isolates. The data thus indicate that the KPC Ω-loop mutations impose a major evolutionary disadvantage toward imipenem/relebactam resistance development. On the other hand, our experimental model also reflects that relebactam exerts a protective effect on the development of resistance to imipenem alone in strains carrying KPC Ω-loop variants, reducing not only the speed at which resistance emerges over time but also the final MIC values reached. These findings regarding the protective effect of relebactam over imipenem resistance development are consistent with previous findings using carbapenem susceptible wild-type (PAO1) and mutator *P. aeruginosa* strains (PAO Δ*mutS*), as researches observed that imipenem/relebactam was clearly superior to imipenem in terms of resistance development kinetics and final levels of resistance reached (17).

### Antibiotic susceptibility and differentially selected resistance mechanisms of mutants selected in the presence of imipenem or imipenem/relebactam

The results of the comparative phenotypic and genomic analysis of the parental isolates and derived lineages evolved in the presence of imipenem or imipenem/relebactam are shown in Table 2 (ST512/KPC-31) and Table 3 (ST258/KPC-35). Considering carbapenems and carbapenem/β-lactamase inhibitor combinations, the evolved mutants developed specific phenotypic and genotypic traits depending on the agent used for selection and independently of the strain considered. In this regard, in all mutants that emerged in the presence of imipenem, carbapenem resistance increased by between 256- and >2,048-fold for imipenem and by 8- to >16-fold for meropenem, resulting in a high-level of cross-resistance for both agents (MICs ranging from 32 to >256 mg/L for imipenem and 32 to >64 mg/L for meropenem). Interestingly, in all imipenem-selected lineages, the addition of relebactam and vaborbactam decreased the carbapenem MICs to values below or slightly above the clinical breakpoint for imipenem/relebactam and meropenem/vaborbactam (MICs ranging from 4 to 16 mg/L for both combinations), thus clearly showing the major role of β-lactamases in these evolved phenotypes. Regarding the ceftazidime/avibactam combination, imipenem-selected mutants from the ST512/KPC-31 lineage maintained or increased resistance levels relative to the parental strain, whereas the ST258/KPC-35 lineages tended to reduce the ceftazidime/avibactam MICs. Different cross-lineage effects on other β-lactams or non-β-lactam agents were not observed.

Further genomic-level examination of the specific mutations presents in mutants obtained under imipenem selective pressure revealed that 12 out of 12 mutants had acquired genetic alterations related to genes coding for KPC enzymes and associated with restoration of the carbapenem-resistant phenotype. Interestingly, in all cases, the KPC-31/ST512 maintained the gene coding for the ancestral KPC-31 β-lactamase enzyme but also developed mutated copies, leading to the replacement of the tyrosine in position 179 in four cases by an aspartic acid and in two cases by an asparagine. These changes resulted respectively in the simultaneous production of a KPC-31 in combination with a natural KPC-3 carbapenemase or a D179N variant, the latter previously obtained in *vitro* by site-directed mutagenesis or in clinical variants, such as KPC-51 (5, 18). Similarly, imipenem-selected mutants of the parental ST258/KPC-35 lineage acquired amino acid substitutions at amino acid position 169, giving rise in four cases to a previously undescribed L169A variant, and in two cases to a restoration of the natural leucine present in the KPC-2 carbapenemase. These data add further evidence of the plasticity of KPC enzymes and are consistent with previous reports showing that carbapenem therapies can select for mutations in KPC Ω-loop variants that restore the natural carbapenemase activity of the KPC enzyme (9).

On the other hand, mutants arising in the presence of imipenem/relebactam acquired distinctive phenotypic and genotypic traits that were to some extent shared across lineages but differed significantly from those observed in mutants selected after imipenem exposure. Except for lineages 3.1 and 3.2 from the ST258/KPC-35 strain, the other mutants developed lower levels of resistance to imipenem and meropenem, which ranged from 2 to 32 mg/L for imipenem (16- to 256-fold MIC increase) and from 16 to 32 mg/L for meropenem (four- to eight-fold MIC increase). While these findings highlight that between carbapenem effects also occurred, in contrast to those mutants selected with imipenem alone, the addition of relebactam or vaborbactam did not significantly potentiate the activity of the respective carbapenem, with most mutants only yielding two- or four-fold reductions in the MICs. The lack of enhanced activity by addition of β-lactamase inhibitors suggests the preponderance of β-lactamase-independent mechanisms in these resistance phenotypes. Conversely, the ceftazidime/avibactam MICs usually increased with most of the mutants tested. In contrast to observations with mutants selected with imipenem, genomic analysis of these imipenem/relebactam-selected lineages revealed that only two out of 12 had acquired mutations in the genes coding for KPC enzymes. These findings clearly demonstrate that relebactam overrides the KPC Ω-loop variant enzyme as an evolutionary advantage toward imipenem resistance, and they explain the observed effects on the resistance development dynamics and the final MIC values for these mutants. Interestingly, the only successful mutant with a KPC substitution had developed a previously undescribed L169T change of unknown effects but putatively associated with the highest imipenem/relebactam MIC values.

Beyond KPCs, the outer membrane porin OmpK36 played a very prominent role in the acquisition of resistance to both imipenem and imipenem/relebactam, as it was disrupted in almost the whole set of mutants sequenced (22/24). This confirms previous observations regarding the key importance of outer membrane permeability on the evolution of resistance toward classic carbapenems but also to the newly developed imipenem/relebactam and meropenem/vaborbactam combinations (19). In most cases, the mutants obtained with imipenem or imipenem/relebactam had also developed mutations in other loci, such as non-coding RNAs (*e.g.*, CopA-like), genes coding for proteins associated with mobile elements (*e.g.*, IS5-like transposase) or structural and metabolic functions (*e.g.*, HtpX, PurR, RfbA, YqjA, GadA). However, the effects on β-lactam resistance or their potential as compensatory mutations are much less predictable and should be analyzed in specific studies.

### Impact of KPC variants selected *in vitro* on β-lactam resistance, laboratory detection and carbapenemase activity

In order to determine the impact of the mutations in the KPC enzymes selected under imipenem or imipenem/relebactam exposure on β-lactam resistance, the genes coding for *bla_KPC-31_*, *bla_KPC-3_*, *bla_KPC-3 D179N_*, *bla_KPC-35_*, *bla_KPC-2_*, *bla_KPC-2 L169A_*, and *bla_KPC-2 L169T_* were cloned in pUCP24 and expressed in the OmpC- and OmpF-deficient *E. coli* HB4 strain. Comparative MIC data for recombinant isolates expressing the parental KPC-31, KPC-35, and the respective derivatives are shown in Table 4. In accordance with the phenotype of the parental strains, expression of KPC-31 and KPC-35 conferred clinical resistance to ceftazidime/avibactam but susceptibility to imipenem, imipenem/relebactam, meropenem and meropenem/vaborbactam. As expected, expression of the natural KPC-2 and KPC-3 enzymes gave susceptibility to ceftazidime/avibactam, imipenem/relebactam and meropenem/vaborbactam, but resulted in a broader substrate spectrum and increased levels of resistance toward classic β-lactams, significantly increasing the MICs of piperacillin/tazobactam, aztreonam, cefepime and carbapenems. Altogether, these findings suggest that selection of a natural KPC variant with carbapenemase activity as a probable evolutionary pathway toward carbapenem resistance in KPC Ω-loop mutants targeted with carbapenems in monotherapy.

**TABLE 4:** Antimicrobial susceptibility profile and performance of diagnostic tests for *E. coli* HB4 strains expressing *bla*_KPC-31_, *bla*_KPC-35_, and the derivatives identified in the isolates evolved in the presence of imipenem and imipenem/relebactam

Strain	MIC (mg/L)^*a*^									Carbapenemase detection^*b*^		
	PT (R > 8)	AZT (R > 4)	CAZ (R > 4)	CZA (R > 8)	FEP (R > 4)	IMI (R > 4)	IMR (R > 2)	MEM (R > 8)	MV (R > 8)	Biochemical test β-Carba	Immunoenzymatic test CORIS BioConcept Resist-5 O.K.N.V.I.	Molecular test Xpert Carba-R
*E. coli* HB4	4	≤0.5	1	0.5	≤0.5	0.125	≤0.06	≤0.06	≤0.06	-	-	-
*E. coli* HB4 KPC-31 (KPC-3 D179Y)	16	4	>64	32	32	0.25	0.125	0.5	0.125	-	+	+
*E. coli* HB4 KPC-3	>512	256	>64	2	128	16	0.125	>64	0.25	+	+	+
*E. coli* HB4 KPC-3 D179N	16	8	>64	32	16	0.25	0.125	0.25	≤0.06	-	+	+
*E. coli* HB4 KPC-35 (KPC-2 L169P)	64	16	>64	16	64	0.25	0.125	2	0.25	-	+	+
*E. coli* HB4 KPC-2	>512	512	>64	2	256	32	0.25	>64	0.5	+	+	+
*E. coli* HB4 KPC-2 L169A	>512	64	>64	8	64	8	0.125	16	0.25	+	+	+
*E. coli* HB4 KPC-2 L169T	>512	128	>64	16	128	16	0.5	16	0.25	+	+	+

^*a*^ 2024 EUCAST breakpoints for Enterobacterales indicated (v. 14.0). PT, piperacillin/tazobactam; AZT, aztreonam; CAZ, ceftazidime; CZA, ceftazidime/avibactam; FEP, cefepime; IMI, imipenem; IMR, imipenem/relebactam; MEM, meropenem; MV, meropenem/vaborbactam.

^*b*^ +, positive test result; -, negative test result.

On the other hand, cloning of the KPC-31-derived D179N variant conferred the same phenotypic pattern of β-lactam resistance as KPC-31. This variant was previously characterized at biochemical and structural levels and has been noted to confer ceftazidime/avibactam resistance through disruption of key stabilizing hydrogen bonds in the Ω-loop, resulting in enhanced catalytic efficiency toward ceftazidime (20). Although this variant did not confer carbapenem resistance, it has been associated with higher imipenem MICs than the D179Y variant (13). It is thus tempting to suggest that this mutation may lead to higher carbapenem MIC values than D179Y when expressed in the genetic background of a clinical strain with additional carbapenem resistance mutations (e.g., total inactivation of OmpK36), explaining its potential emergence upon imipenem exposure. However, the most concerning phenotypic resistance profile was probably that observed for the transformants expressing the L169A and L169T variants, selected with respectively imipenem and imipenem/relebactam and showing a hybrid antimicrobial susceptibility profile between those of KPC-2 and KPC-35, associated with resistance to both ceftazidime/avibactam and carbapenems. Interestingly, combined resistance to ceftazidime/avibactam and carbapenems has already been observed in clinical strains carrying ceftazidime/avibactam-resistant KPC variants with substitutions at position L169 other than proline, such as KPC-12 (L169M) or KPC-62 (L169Q) (21, 22). In accordance with the observations on the restoration of carbapenem resistance, these L169 variants were the only ones able to confer ceftazidime/avibactam resistance and also to yield a positive hydrolysis result in the biochemical detection tests evaluated. The impact on detection with immunoenzymatic tests or PCR-based approaches was not observed for any of the variants cloned, suggesting weak effects of changes in positions L169 or D179 on the effectiveness of these diagnostic methodologies to detect KPC enzymes.

Subsequent biochemical assays with the different KPC variants confirmed that the amino acid changes associated with the restoration of a carbapenem-resistant phenotype in the cloning experiments resulted in a significant increase in the specific activity of the KPC enzymes toward imipenem (Table S1). On the other hand, comparative analysis of the inhibitory activity of avibactam and relebactam against the different variants did not reveal important differences with respect to the parental enzymes, although the KPC-35-derived variants were inhibited at slightly lower concentrations by both diazabicyclooctanes than the parental KPC-35 enzyme. Altogether, these data confirm that the evolutionary pathway of these KPC Ω-loop mutants toward carbapenem resistance results from substitutions that enhance their ability to hydrolyze carbapenems without significant collateral effects on their susceptibility to avibactam or relebactam.

### Conclusions

The study findings conclusively demonstrates that, relative to imipenem alone, the imipenem/relebactam combination suppresses the appearance of resistant mutants at low concentrations, delays the development of resistance, and reduces the levels of resistance reached *in vitro* against clinical strains of *K. pneumoniae* carrying KPC Ω-loop variants associated with ceftazidime/avibactam resistance. We also show that this is mechanistically explained by the drastic counter-effect that the imipenem/relebactam combination has on the evolution of KPC enzymes, as the carbapenem unprotected rapidly favors the selection of strains carrying multiple KPC variants or associated with hybrid phenotypes that limit the effectiveness of both carbapenems and ceftazidime/avibactam. Thus, although further *in vivo* data and clinical experience must be obtained, our data position imipenem/relebactam as a promising therapeutic option for combating ceftazidime/avibactam-resistant KPC-producing *K. pneumoniae* infections.

## MATERIALS AND METHODS

### Clinical strains

Two ceftazidime/avibactam-resistant *K. pneumoniae* isolates recovered from patients previously treated with ceftazidime/avibactam and showing distinctive Ω-loop amino acid substitutions and belonging to high-risk sequence types were selected for the study: KPC-35 (KPC-2 L169P)/ST258 and KPC-31 (KPC-3 D179Y)/ST512. Both isolates displayed collateral imipenem and imipenem/relebactam susceptibility. These strains were chosen as they mirror the main lineages that circulate with KPC-2 and KPC-3 enzymes worldwide, are prone to accommodating KPC substitutions associated with ceftazidime/avibactam resistance, and represent potential targets for both imipenem and imipenem/relebactam therapies.

### Single-step mutant frequencies and mutant prevention concentrations

For each parental strain, approximately 5 × 10^8^ cells from fresh Mueller–Hinton (MH) broth cultures were respectively transferred to MH agar plates containing incremental concentrations of imipenem or imipenem/relebactam up to 64 mg/L (relebactam was added at a fixed concentration of 4 mg/L). The plates were incubated for 48 h at 37°C before the frequency of resistance emergence was calculated as the ratio of the number of colony forming units (CFUs) grown on the antibiotic-containing plate and the number of CFUs on antibiotic-free plates. Mutant prevention concentrations were defined as the lowest concentration of antibiotic combination for which there were zero colonies on all agar plates (23). All determinations were conducted in triplicate.

### *In vitro* resistance dynamics

Resistance dynamics were determined following the protocol of our previously described methodology (16). On day 0, 10 mL of MH broth tubes containing 0.5×, 1×, 2×, 4×, 8×, 16×, 32×, and 64× MIC concentrations of imipenem or imipenem + 4 mg/L of relebactam were inoculated with each of the two parental strains (10^6^ CFU/mL concentration) in triplicate experiments. The tubes were then incubated for 24 h at 180 rpm at 37°C, and those with the highest antibiotic concentration showing visible growth were reinoculated at a 1:1,000 dilution ratio in fresh tubes containing incremental concentrations up to 64× MIC for seven consecutive days. Two colonies per strain, antibiotic, final resistance step, and replicate experiment were isolated in antibiotic-free MH agar plates for further characterization.

### Antimicrobial susceptibility testing

The MICs of piperacillin/tazobactam, aztreonam, ceftriaxone, ceftazidime, ceftazidime/avibactam, cefepime, ertapenem, imipenem, meropenem, tigecycline, trimethoprim/sulfamethoxazole, ciprofloxacin, levofloxacin, gentamicin, tobramycin, and amikacin were determined in triplicate experiments by broth microdilution in Sensititre plates (GN6F Sensititre panels, Thermo Fisher Scientific, USA). The MICs of imipenem, imipenem/relebactam, meropenem, meropenem/vaborbactam, ceftazidime, and ceftazidime/avibactam were also determined in triplicate experiments using the reference broth microdilution method. The MICs were interpreted using EUCAST 2024 breakpoints v. 14.0 (www.eucast.org/clinical_breakpoints).

### Whole genome sequencing and resistance genomics

Both parental strains and also two mutant isolates per strain, antibiotic, final resistance step, and replicate experiment were sequenced in an Illumina NovaSeq6000 (Illumina Inc, CA). Total genomic DNA was obtained using a Genomic DNA Buffer Set with a Genomic-tip 20/G (QIAGEN). The Illumina indexed paired-end libraries were generated from a Nextera DNA XT Library Prep Kit (Illumina). Reads were quality controlled with fastp (v. 0.23.2) (24) and assembled with Unicycler (v. 0.5.0) (25). The resulting assemblies were assessed with CheckM (v. 1.2.0) (26), ensuring 100% completeness, <1% contamination, and 0% heterogeneity in all cases, with an average median depth over 300×. MLST identification was performed through mlst (v. 2023–03) (27), while resistance determinants were analyzed using ResFinder (v. 4.1.5) (28) and CARD (v. 3.2.8) (29). Reference genome *K. pneumoniae* ATCC 10031 and parental strains (ST512/KPC-31 and ST258/KPC-35) were annotated through bakta (v. 1.7.0) (30). Snippy (v. 4.6.0) (31) was then used for variant calling of each lineage against both ATCC 10031 and their respective parental strain using their reads, with a minimum depth of 5× and allowing heterogeneous positions (e.g., positions where 50% of reads indicate a specific nucleotide, while the rest indicate a different one). These positions were manually reviewed using read alignments through the Integrative Genomics Viewer (IGV) (32) and the assembly graphs with Bandage (33), facilitating the detection of multiple KPC alleles in specific strains.

### Molecular cloning

The *bla*_KPC-35_, *bla*_KPC-31_, and the *bla*_KPC_ alleles identified in imipenem or imipenem/relebactam-selected mutants were amplified in parallel with the primer pair KPC-F-SacI (5´-CGAGCTCGCCATGCCCATATCCTGACCCTG-3´) and KPC-R-BamHI (5´-CGGGATCCGCCGCGCAGACTCCTAGCCTAAA-3´), ligated to the pUCP24 plasmid, and cloned into porin-deficient *Escherichia coli* HB4 (low-permeability model), as previously described (34). Recombinant strains were selected in 10 mg/L of gentamicin-LB agar plates. The β-lactam MICs were determined for the transformants according to the above-described methodology.

### Detection of KPC β-lactamases and carbapenemase activity

The KPC β-lactamases were detected with the recombinant *E. coli* HB4 isolates expressing the different *bla*_KPC_ genes in the pUCP24 plasmid. The carbapenemase activity was determined using the β-Carba test (Bio-Rad, Marnes-la-Coquette, France) (35). Immunoenzymatic detection was performed using the Resist-5 O.K.N.V.I. assay (CORIS BioConcept, Gembloux, Belgium) (36), and the molecular detection was carried out using the Xpert Carba-R kit (Cepheid, Sunnyvale, CA, USA) (37). All commercial methods were performed following manufacturers’ instructions.

### Enzyme kinetic measurements of KPC enzymes

Crude extracts of *E. coli* HB4 strains expressing *bla*_KPC-31_, *bla*_KPC-35_, and derivatives identified in isolates evolved in the presence of imipenem and imipenem/relebactam were obtained by growing an overnight culture in 500 mL of Luria–Bertani broth containing 10 mg/L of gentamicin at 37°C (7, 38) . Cultures were centrifuged at 3,500 × *g*, 15 min at 4°C, resuspended in 10 mL of phosphate-buffered saline (PBS; pH ~7.4) and sonicated with Ultraschall Prozessor UP 200S Hielscher (Teltow, Germany). The sonicate was centrifuged at 11,000 × *g*, 1 h at 4°C. β-Lactamases from crude extracts were used to measure the specific activity against imipenem with a Specord 200 Plus spectrophotometer (Analytic Jena, Thuringia, Germany) and the 50% inhibitory concentration (IC_50_) of avibactam and relebactam using an EPOCH 2 microplate spectrophotometer (Biotek, VT, USA). A wavelength of 297 nm and an absorption coefficient of 29,210 M^−1^ cm^−1^ were used for imipenem in the determination of the specific activity. The IC_50_ parameter was defined as the inhibitor concentration required to reduce the hydrolysis rate of nitrocefin by 50%, after preincubation of the crude extract with different concentrations of the inhibitors, at room temperature for 10 min.

## SUPPLEMENTAL MATERIAL

Table S1 aac.01120-24-s0001.docx Specific β-lactamase activity against imipenem and IC50s for avibactam and relebactam toward KPC enzymes.

ASM does not own the copyrights to Supplemental Material that may be linked to, or accessed through, an article. The authors have granted ASM a non-exclusive, world-wide license to publish the Supplemental Material files. Please contact the corresponding author directly for reuse.
