# Supplementary material for: Mutant prevention concentrations, in vitro resistance evolution dynamics, and mechanisms of resistance to imipenem and imipenem/relebactam in carbapenem-susceptible Klebsiella pneumoniae isolates showing ceftazidime/avibactam resistance
Source: Antimicrob Agents Chemother. 2024 Nov 15;68(12):e01120-24. doi: 10.1128/aac.01120-24 (PMC11619344; doi:10.1128/aac.01120-24)
Supplement: Table S1 — Specific β-lactamase activity against imipenem and IC50s for avibactam and relebactam toward KPC enzymes. [file aac.01120-24-s0001.docx]

| **Table S1.**  Specific β-lactamase activity against imipenem and IC_50_s for avibactam and relebactam towards KPC enzymes. | | | | |
| --- | --- | --- | --- | --- |
| Strain | Phenotype | Specific activity  (µmol·min^-1^·mg^-1^) | IC_50_ AVI  (µM) | IC_50_ REL  (µM) |
|  |  | IMI |  |  |
| KPC-31 (KPC-3 D179Y) | ESBL | ND | 0.05 | 0.03 |
| KPC-3 | Carbapenemase | 2.5 | 0.03 | 0.07 |
| KPC-3 D179N | ESBL | 0.02 | 0.1 | 0.05 |
| KPC-35 (KPC-2 L169P) | ESBL | ND | 0.5 | 0.3 |
| KPC-2 | Carbapenemase | 0.55 | 0.02 | 0.03 |
| KPC-2 L169A | Carbapenemase | 0.1 | 0.06 | 0.1 |
| KPC-2 L169T | Carbapenemase | 0.13 | 0.02 | 0.04 |
| ESBL: extended-spectrum β-lactamase; IMI: imipenem; AVI: avibactam; REL: relebactam; ND: not detectable. | | | | |
